# Questionnaires for the Assessment of Central Sensitization in Endometriosis: What Is the Available Evidence? A Systematic Review with a Narrative Synthesis

**DOI:** 10.1007/s43032-023-01343-4

**Published:** 2023-09-26

**Authors:** Giulia Emily Cetera, Camilla Erminia Maria Merli, Giussy Barbara, Carlotta Caia, Paolo Vercellini

**Affiliations:** 1 Gynecology Unit, Fondazione IRCCS Ca’ Granda Ospedale Maggiore Policlinico, Milan, Italy; 2 Department of Clinical Sciences and Community Health, University of Milan, Milan, Italy; 3 Obstetric and Gynecological Emergency Unit and SVSeD (Service for Sexual and Domestic Violence), Fondazione IRCCS Ca’ Granda Ospedale Maggiore Policlinico, Milan, Italy

**Keywords:** Endometriosis, Central sensitization, Central sensitization scale, Chronic pelvic pain

## Abstract

It has been suggested that central sensitization (CS) may be involved in the failure of standard medical and surgical treatment to relieve endometriosis-related pain. However, there is no gold standard for the diagnosis of CS, and self-reported questionnaires are used as diagnostic surrogates. The main objective of this review was to identify all CS questionnaires used in clinical endometriosis studies. The secondary objective was to qualitatively analyze strengths and weaknesses of each questionnaire. A PubMed and EMBASE systematic literature search conducted in April 2023 using the terms “endometriosis; central pain; central sensitization; questionnaire; patient-reported outcome measure; screening tool” identified 122 publications: six articles were included in the review. The Central Sensitization Inventory (CSI) is the most frequently used questionnaire for the detection of CS in patients with endometriosis. It has been validated in patients with endometriosis, in whom it appears to have good psychometric proprieties. The Fibromyalgia Survey Questionnaire (FSQ) has also been used, although it has not been specifically validated in endometriosis patients. The debate regarding these questionnaires’ construct validity is still open and will be so until a gold standard diagnostic tool for CS is found. In fact, some authors argue these questionnaires are measuring psychological vulnerability and a hypervigilant state that is associated with pain, rather than CS itself. However, their use should not be discouraged as they are able to identify chronic pain patients which warrant further attention and who may benefit from broader treatment strategies.

**Supplementary Information:**

The online version contains supplementary material available at 10.1007/s43032-023-01343-4.

## Introduction

Conventional medical and surgical therapies fail to relieve endometriosis-related pain in up to one-third of patients [1]. It has been suggested that modifications in the functioning of the central nervous system, which alter the perception of pain and are known under the name of central sensitization (CS), may be involved in such phenomenon [2, 3].

The precise mechanisms through which CS develops are yet to be understood, although adaptive pre-synaptic (changes in neurotransmitter release) and post-synaptic modifications (changes in the activity of post-synaptic channels) in response to reiterative peripheral signaling seem to play a role [4]. However, authors have found an agreement regarding CS’ core features, which appear to be the result of the lowering of thresholds for central neuronal activation and include hyperalgesia, allodynia, enlargement of receptive field size, and maintenance of pain once the painful stimulus has ceased. A predisposition to the development of fatigue, depression, and other chronic pain conditions has also been observed in patients with CS [5, 6].

Studies using quantitative sensory testing (QST) have indeed shown that patients with endometriosis have significantly altered pain thresholds both in endometriosis sites and in other locations which are not related to the disease [7, 8]. Moreover, endometriosis has been included in the National Institutes of Health Pain Consortium list of Chronic Overlapping Pain Conditions (COPCs), a set of chronic pain conditions which often co-occur in the same individual and appear to share CS as a common underlying mechanism [9].

Accordingly, evidence regarding the role of CS in patients’ response to standard treatments for endometriosis is increasing [10, 11]. In fact, higher measures of CS are associated with a reduced response both to surgical and medical therapy [10–12]. However, such correlation may be read in either direction: as CS predicting worse outcomes and as worse outcomes leading to a reduced improvement of CS measures following treatment. What is clear is that CS and response to conventional treatment are strictly intertwined and as such identifying patients with endometriosis whose pain may have a significant central component is crucial.

A multidisciplinary care program based on a biopsychosocial approach including pain education, cognitive behavioral therapy, pelvic muscle physical therapy, and targeted central pharmacology has been suggested for the management of CS [13] and is highly encouraged as it may represent the missing piece in the treatment of endometriosis-related pain. Moreover, in patients with more than one COPC, the management and treatment of all coexisting painful conditions are suggested as they have been shown to improve clinical outcomes [9].

However, although the presence of comorbid COPCs may be ruled out by referring patients to corresponding specialists, there is no gold standard for the diagnosis of CS. Quantitative sensory testing, neuroimaging techniques, and somatosensory-evoked potentials are used in research settings but are not easily applicable to clinical practice. For this reason, self-reported questionnaires are used as diagnostic surrogates [14, 15].

The main objective of this review was to identify all CS questionnaires described in the literature and used in clinical endometriosis studies. The secondary objective was to qualitatively analyze strengths and weaknesses of each questionnaire.

## Methods

This systematic review was carried out following the Preferred Reporting Items for Systematic Reviews and Meta-Analyses (PRISMA) indications [16]. The complete PRISMA checklist is provided (Supplemental Table 1). Not every item of the checklist could be applied to our review, as a qualitative approach was used to summarize the data.

A PubMed and EMBASE systematic literature search was conducted in April 2023 (last search conducted on April 23^rd^, 2023) using the terms “endometriosis; central pain; central sensitization; questionnaire; patient-reported outcome measure; screening tool.” No time restrictions were applied. Abstracts and papers not written in English were excluded along with articles not reporting original data. Observational, retrospective, and prospective studies, controlled clinical trials, and RCTs were included in the research.

Two authors (G. E. C. and C. E. M. M.) assessed the papers and independently selected the articles considered eligible for the review. Publications were included if they analyzed CS in patients with endometriosis using a specific questionnaire. Reference lists were checked to identify other potentially relevant studies. Discrepancies were resolved by discussion. Data extraction was performed independently by G. E. C. and C. E. M. M., who retrieved information regarding authors, date, and country of publication, study design and methods, study population, type of CS questionnaire, objectives, and results. Extracted information was organized in an Excel spreadsheet. No attempt was made to retrieve unpublished material.

A qualitative analysis was performed to analyze the papers included in the review. The questionnaires were described and compared using Bourdel’s criteria for the assessment of pain scales in endometriosis [17]. These are a set of nine criteria previously published by the IMMPACT group (Initiative on Methods, Measurement and Pain Assessment in Clinical Trials) [18], the Art and Science of Endometriosis meeting [19], and the FDA [20] and adapted by Bourdel and co-workers to the specificity of endometriosis. The criteria include (1) scale description and application; (2) validity, reliability/reproducibility, and responsiveness; (3) disease specificity and multidimensionality; (4) respondent and investigator burden and feasibility; (5) validation in foreign languages; (6) precise pain measurement and pain measurement inclusion criteria; (7) timing of pain assessment; (8) PRO and PRO instrument; and (9) responder concept and minimal clinically important difference after treatment (MCID).

Data regarding the assessment of CS through specific questionnaires in other specialties was also used when deemed relevant for comparison.

## Results

**Fig. 1:**
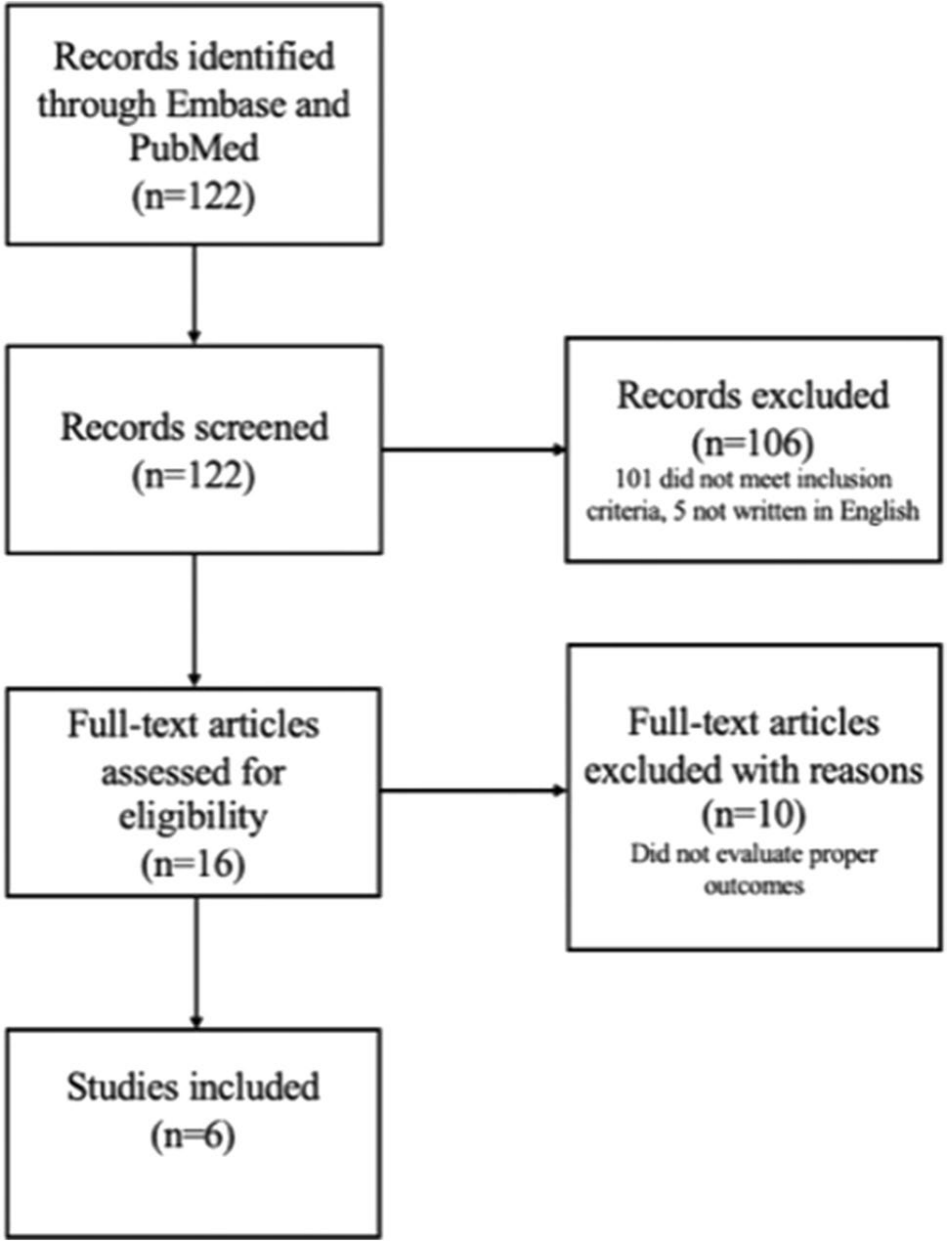
Flowchart

A total of 122 publications were identified on PubMed and EMBASE. Following abstract screening, 106 articles were excluded (five were not written in English, 101 did not meet inclusion criteria). Among the 16 which were considered eligible for in-depth reading, ten were excluded because they did not evaluate proper outcomes [14, 21–29], leaving six articles deemed eligible for the review [5, 6, 10–12, 30]. The flowchart of the selection process of the included studies is represented in Fig. 1.

The questionnaires measuring CS used in the six articles included in the review were the Central Sensitization Inventory (CSI) and the Fibromyalgia Survey Questionnaire (FSQ). Below we provide a brief description of each questionnaire as well as a comparative analysis of the two. An overview of the included studies is provided in Table 1.

**Table 1:** Articles included in the review

	Author, year	Study type	Study population	CS measure	Objectives	Results
1	Orr et al. 2019	Cross-sectional analysis	163 women with endometriosis	CSI	Comparison of CSI scores among three subgroups of patients: (1) patients with no/low deep dyspareunia; (2) patients with high deep dyspareunia and no bladder-pelvic floor tenderness (BPFT); (3) patients with high deep dyspareunia and BPFT	The CSI was higher in women with deep dyspareunia and BPFT compared with women with no or low deep dyspareunia (*p* < 0.001) and compared with women with high deep dyspareunia but no BPFT (*p* = 0.001). 74% of the subgroup with high deep dyspareunia and BPFT had a CSI score greater than cutoff, compared to 53% of the subgroup with high deep dyspareunia and no BPFT and to 27% of the subgroup with no or low deep dyspareunia
2	As-Sanie et al. 2022	Prospective, observational study	126 women with chronic pelvic pain (CPP) (including endometriosis-related CPP)	FSQ	To describe the incidence of persistent pelvic pain (failure to achieve 50% or more improvement in average pelvic pain score) in the 6 months following hysterectomy in women with CPP and to determine whether CS is associated with greater risk of persistent pelvic pain following surgery	Fifteen (11.9%) patients reported persistent pelvic pain six months after hysterectomy, which was performed for abnormal uterine bleeding, CPP, dysmenorrhea, fibroids or prolapse. Every 1-point increase in centralized pain prior to hysterectomy was associated with a 27% increase in the odds of persistent pelvic pain (OR 1.27, 95% CI 1.03–1.57) six months after hysterectomy. These findings held true across the continuum of the FSQ, even among patients who did not meet the criteria for fibromyalgia diagnosis (defined as a score of 13 or more)
3	Orr et al. 2022	Proof of proof concept study	335 women with endometriosis	CSI	Identify a CSI cutoff in the endometriosis population, to discriminate between individuals with significant central contributors (identified by ≥ 3 CSS) to their pain and those without	An increasing number of CSS was significantly correlated with dysmenorrhea, deep dyspareunia, dyschezia and chronic pelvic pain scores (*p* < 0.001) and with the CSI score (*p* < 0.001). 151/335 (45%) of patients had a low (< 40) CSI score and 184/335 (55%) had a high (≥ 40) score A CSI cutoff of 40 had a sensitivity of 78% (95% CI 72.7–84.6%) and a specificity of 80% (95% CI 70.3–84.5%) for identifying a patient with endometriosis with ≥ 3 CSS. In the group with CSI ≥ 40, 18% self-reported pain non-responsive to hormonal therapy and 40% reported daily pain, compared with 6% and 20% in the CSI < 40 group (*p* = 0.003 and 0.002, respectively)
4	Orr et al. 2023	Prospective, longitudinal cohort study	239 patients with diagnosed or suspected endometriosis who underwent surgery	CSI	Association between CSI baseline scores and persistent pain after endometriosis surgery (both conservative and hysterectomy)	Higher baseline CSI scores were significantly associated with higher chronic pelvic pain (OR 1.02; 95% CI 1.00–1.03; *p* = 0.02); deep dyspareunia (OR 1.03; 95% CI 1.01–1.04; *p* = 0.04); dyschezia (OR 1.03; 95% CI 1.01–1.04; *p* = 0.04) and back pain (OR 1.02; 95% CI 1.00–1.03; *p* = 0.02) at follow-up, following surgery. CSI scores themselves decreased slightly from baseline to follow-up (mean score 43.8 vs 41.7, *p* = 0.05). The significant association between the number of CSS and increasing CSI scores was significant both at baseline (*p* < 0.001) and at follow-up (*p* < 0.001)
5	Quintas-Marquès et al. 2023	Cross-sectional study	160 consecutive premenopausal women with recently suspected DE	CSI	To assess the prevalence of CS-related symptoms in patients with DE compared to HCs and to analyze the clinical characteristics of patients with DE and CS-related symptoms	Women in the DE group reported higher CSI scores (*p* = 0.015), more pain symptoms (*p* < 0.001), lower quality of life, as measured with the SF36 questionnaire (*p* < 0.001), and higher scores of depression and anxiety, measured with the HADS scale (*p* < 0.001), compared to the HC group. Comparing women with DE and a CSI score ≥ 40 with those with DE and a CSI score < 40, the former had higher scores in pain symptoms (*p* < 0.001), poorer quality of life (*p* < 0.001) and a higher risk of depression and anxiety (*p* < 0.001)
6	Raimondo *et al*, 2023	Observational cross-sectional study	285 consecutive women with endometriosis	CSI	CS prevalence and associations between demographic and clinical factors and CS	CS prevalence was 41.4% (95% CI 35.8–47.2). There was a significant association between CS and moderate-to-severe chronic pelvic pain, postero-lateral parametrium involvement, hormonal therapy failure (persistence of at least one moderate-severe pain symptom after at least 3 months of medical therapy), migraine or tension type headache, irritable bowel syndrome, anxiety and panic attacks

*BPFT* bladder-pelvic floor tenderness, *CPP* chronic pelvic pain, *CSI* Central Sensitization Inventory, *CSS* central sensitivity syndromes, *DE* Deep endometriosis, *FSQ* Fibromyalgia Survey Questionnaire, *HADS* Hospital Anxiety and Depression Scale, *HC* healthy controls, *SF-36* Short-Form 36-item Survey

### Central Sensitization Inventory

The CSI was used to measure CS in patients with endometriosis in five out of the six studies included in the review [5, 6, 11, 12, 30].

This questionnaire was designed to quantify the degree of COPC-related symptoms, in order to establish the level of CS impairment among patients suffering from chronic pain. It was initially validated in fibromyalgia patients [31] and was subsequently also validated in patients with endometriosis [6].

The CSI is a self-reported measure, which is divided in two parts (A and B). Part A assesses 25 symptoms, each of which is measured by the means of a Likert scale (0, never; 1, rarely; 2, sometimes; 3, often). Scoring ranges from 0 to 100 with a cutoff point ≥ 40 considered the threshold of clinical relevance [32]. Higher scores are associated with a higher degree of CS [33]. Part B investigates whether the patient has previously been diagnosed with one or more specific conditions, including seven COPCs (tension headache or migraine, irritable bowel syndrome (IBS), fibromyalgia, restless leg syndrome, temporomandibular joint disorder, chronic fatigue syndrome, and multiple chemical sensitivity) and three CS-related disorders (depression, anxiety or panic attacks, and neck injury). Four of the ten COPCs identified by the National Institutes of Health Pain Consortium (vulvodynia, endometriosis, painful bladder syndrome, chronic low back pain) are not included in part B of the questionnaire.

CSI is not a neurophysiological measure and thus not a direct marker of CS [6]; however, it is able to discriminate between patients with COPCs and patients with chronic pain conditions without a central component of pain, as well as between patients with COPCs and healthy controls [31, 32].

### Fibromyalgia Survey Questionnaire

In one study, CS was measured by the means of the FSQ [10]. This is a validated self-reported measure, which was initially intended to be used as a diagnostic tool for fibromyalgia. However, owing to the hypothesis that fibromyalgia may represent the extreme end of a continuous spectrum of a polysymptomatic distress condition in which CS plays a key role, the FSQ has also been suggested as a proxy index for CS [34]. For this reason, the FSQ has also been called “fibromyalgianess scale,” “central sensitivity score,” and “polysymptomatic distress scale” [35].

The questionnaire is the sum of the Widespread Pain Index, i.e. the total number of painful body areas (0 to 19 points), and of the Symptom Severity Scale, that is, the severity of related symptoms such as fatigue, trouble thinking, sleeping difficulties, pain or cramps in the lower abdomen, headache, and depression (0 to 12 points). The total score may range from 0 to 31 points, with scores ≥ 13 considered indicative for fibromyalgia. Conversely, when applied for the evaluation of central pain, the FSQ is used as a continuous measure, with higher values indicating a greater degree of CS [36].

### Comparative Analysis of CSI and FSQ

We provide a brief description and comparison of the psychometric proprieties of the abovementioned questionnaires.

#### Questionnaire Description and Application

Both questionnaires have been extensively described in the literature and are easily available for research and clinical use. Unlike the FSQ, the CSI has been validated in patients with endometriosis [6] and as such is the most frequently used tool to measure CS in these patients.

In the five studies using the CSI to measure CS in patients with endometriosis, the questionnaire was used in its original format by all authors, except for Orr and co-workers. In two of their publications [6, 11], instead of using part B of the CSI questionnaire, the authors investigated the presence of comorbid COPCs and pain-related comorbidities using pre-existing screening or diagnostic tools, specific for each disease. These included self-reports of fibromyalgia, chronic fatigue syndrome, and migraines; the Rome III criteria for IBS [37], the American Urology Association [38], or the International Continence Society [39] criteria for painful bladder syndrome; the Carnett test for abdominal wall pain [22]; digital palpation on pelvic examination for myofascial pelvic syndrome [3]; the Patient Health Questionnaire for depressive symptoms [40]; the Generalized Anxiety Disorder criteria for anxiety symptoms; and the Pain Catastrophizing Scale for measuring catastrophizing [41].

A 9-item short form of the CSI part A has been developed and validated in patients with musculoskeletal pain, although to our knowledge it has never been applied in studies on endometriosis [42].

In As-Sanie and co-workers’ study, the FSQ was used in its original format [10]. We are not aware of the presence of a short form of the FSQ.

#### Validity, Reliability/Reproducibility, and Responsiveness

Construct validity is defined as the degree to which an instrument measures the construct that it is supposed to measure. It is measured with the coefficient “r,” which may vary between 0 and 1.00, with higher values indicating greater validity. The validation of a questionnaire may be obtained by comparing it with other validated tools, which measure a similar construct [43]. As such, questionnaires investigating the presence of CS may be compared with those analyzing pain and with validated tools used to diagnose or screen for COPCs.

A considerable number of studies [32, 33, 44–49] have proven CSI’s good construct validity in chronic pain populations (*r* = 0.46–0.73). In endometriosis patients, Orr and co-workers found good construct validity when correlating CSI scores with the number of COPCs (*r* = 0.45–0.73); higher pain scores (*r* = 0.21–0.46); earlier onset of pain (*p* = 0.005); non-response to hormonal treatment (*p* = 0.003); daily pain (*p* < 0.001); and higher pain scores following endometriosis surgery (*p* = 0.02) [5, 6, 11]. Also Raimondo and co-workers found a correlation between CSI scores and higher pain scores (*p* = 0.01); COPCs such as IBS and migraine or tension-type headache (*p* = 0.005–0.008); anxiety (*p* = 0.01); and hormonal treatment failure (*p* = 0.02) [12]. When specifically analyzing women with deep endometriosis, Quintas-Marquès and colleagues found a positive association between CSI and higher pain scores (*p* < 0.001), lower quality of life (*p* < 0.001), and depressive and anxiety symptoms (*p* < 0.001) [30].

However, interpretation of this data should be cautious as a clear explanation of how CS develops and what it entails is yet to be found. As such, correlating CSI scores with pain characteristics without exploring causal pathways may be confounding as pain perception may be enhanced by CS in the same way CS-related symptoms may be a consequence of chronic pain.

To our knowledge, few attempts have been made to evaluate the validity of FSQ as a measure of CS in chronic pain populations. In their study conducted on 1651 patients to analyze the validity of the FSQ as a tool for the identification of fibromyalgia (thus, not as a tool for the detection of CS), Hauser and co-workers found a moderate correlation (*r* = 0.48) between FSQ and the Patient Health Questionnaire-4, a self-report questionnaire for the evaluation of depression and anxiety [50]. Moreover, in patients who had undergone a hysterectomy for benign conditions (the type of condition was not specified) and in those who had been treated with lower-extremity joint arthroplasty, perioperative FSQ scores predicted worse postsurgical pain scores and a greater opioid requirement [51, 52]. Quantitative sensory testing measures of central sensitization also positively correlated with FSQ scores in female patients with knee osteoarthritis [53], as in both male and female patients with rheumatoid arthritis [54].

As-Sanie and colleagues were the first authors to measure CS in women with endometriosis (the study also included women who had undergone a hysterectomy for other benign conditions) using the FSQ. In their study, every 1-point increase in the FSQ prior to hysterectomy was associated with a 27% increase in odds of persistent pain (*p* = 0.026) [10].

CSI’s reliability, which is the extent to which repeated measurements agree with one another [43], has been extensively measured in chronic pain populations in terms of test-retest reliability (intraclass correlation coefficients 0.82–0.97) [31, 32, 48, 55, 56]. However, it has not been analyzed specifically in patients in endometriosis.

Similarly, test-retest reliability of the FSQ has been evaluated in three studies conducted on chronic pain patients and has been found to be good (intraclass correlation coefficient 0.86, 0.79, and 0.71, respectively) [50, 59, 60], while no attempt to measure the reliability of the FSQ when used in patients with endometriosis has been carried out to date.

Responsiveness, i.e., the ability of a measure to detect change over time or following treatment [43], has been proven for CSI both in chronic pain populations [33, 44] and in endometriosis patients [6]. To our knowledge, it has never been analyzed for the FSQ when used to measure CS.

#### Disease Specificity and Multidimensionality

Neither CSI nor FSQ are disease-specific, although the FSQ is also used to diagnose fibromyalgia. In fact, they both analyze a plethora of CS-related symptoms, which may be found in COPCs, in other non-CS related chronic conditions, and in mood disorders as in healthy controls [6, 31, 35]. As such, both questionnaires are multidimensional as they measure physical, psychological, and cognitive functioning and physical symptoms [31, 34].

#### Respondent and Investigator Burden and Feasibility

Both questionnaires are straightforward, relatively short, self-reported scales. This makes them potentially easy to administer in clinical settings or even electronically. To our knowledge, the literature is lacking evidence regarding patient preference in terms of burden and feasibility of the CSI. In Hauser and colleagues’ validation study, the acceptance of the FSQ items ranged between 78.9 and 98.1% [50].

#### Validation in Foreign Languages

The CSI has been translated in 19 different languages and its cross-cultural validity, that is, the degree to which the performance of the items on a translated or culturally adapted version of the questionnaire is an adequate reflection of the original version [43], has been proven by various authors [48, 55, 56].

The FSQ has been translated and cross-culturally validated in six languages [50, 57–62].

#### Precise Pain Measurement and Pain Measurement Inclusion Criteria

Neither the CSI nor the FSQ are direct measures of pain. Rather, they are indirect measures of the central component of pain, and specifically they measure COPCs, which are not quite the same as CS [15]. A cut-off of 40 has been established for the CSI, both in chronic pain patients [32] and in patients with endometriosis [6].

In 2017 Neblett and co-workers established a gradient of clinically relevant severity levels of the CSI: subclinical (score 0 to 29); mild (30 to 39); moderate (40 to 49); severe (50 to 59); and extreme severity (60 to 100) [33]. Cuesta-Vargas and colleagues also identified three severity clusters: low level of CS-related symptom severity; medium level of CS-related symptom severity; and high level of CS-related symptom severity [62]. Clinicians and researchers may easily assess symptom severity according to Cuesta-Vargas’ scale by using a free online calculator, which may be found at https://www.pridedallas.com/questionnaires.

The FSQ does not have a cut-off for central pain as it is used as a continuous measure, with higher values indicating a greater degree of CS [36]. However, to aid statistical analysis in their studies, both As-Sanie and Brummet and colleagues classified their patients in three different severity groups: low (scores from 0 to 4); moderate (scores from 5 to 8), and high severity (scores from 9 to 31) [10, 52].

#### Timing of Pain Assessment

Both CSI and FSQ are easy to comprehend and may be filled out in a relatively short amount of time [63]. As such they may represent a useful aid also during or before clinical practice, helping the physician to recognize patients whose pain has a significant central component and as such may not be entirely responsive to standard treatment.

#### PRO and PRO Instrument

A patient-reported outcome (PRO) is any report of the status of a patient’s health condition which comes directly from the patient, without an interpretation of the patient’s response from a third party [17]. Both CSI and FSQ are PRO instruments, being self-reported questionnaires.

#### Responder Concept and MCID

A patient is considered a responder when researchers are able to detect the smallest score change in a measure, experienced individually, that has been considered in the population to have a significant treatment benefit [17]. More specifically, minimal clinically important difference after treatment (MCID) is defined as “the smallest difference in score in the domain of interest that patients perceive as important, either beneficial or harmful, and that would lead the clinician to consider a change in the patient’s management” [64]. MCID has not been described yet in endometriosis patients, neither for CSI nor for FSQ.

## Discussion

The CSI and the FSQ are the two self-reported questionnaires retrieved in the literature for the measurement of CS in patients with endometriosis. The CSI is the most frequently used, probably due to the fact it has been specifically validated in this population and has been found to have good psychometric proprieties [65].

In the last two decades, the focus of endometriosis treatment has gradually shifted from pathological classification improvement to improvement in patient reportings of pain [17], to patients’ vulnerability to pain [65]. In fact, it is now established that some individuals are characterized by a greater responsiveness to noxious and non-noxious stimuli, probably due to an altered functioning of central synapses [66]. This phenomenon has been defined as “central sensitization” and seems to be applicable to endometriosis-related pain as to pain caused by other chronic pain conditions, named COPCs [9].

The fact that some individuals with endometriosis may be more vulnerable to pain than others entails a series of consequences on clinical practice, which can no longer be overlooked. Firstly, identifying patients with a significant central component of pain enables clinicians to offer these individuals an adequate standard of care, including a multidisciplinary care program for the treatment of CS alongside conventional therapy [13]. Secondly, partial or non-response to standard treatment in patients with and without CS should be interpreted in a different manner. In fact, among those with a greater central component of pain, treatment of peripheral factors may not be sufficient [6]. Thus, suggesting cognitive behavioral therapy, pelvic muscle physical therapy, pain education, acupuncture, and/or targeted central pharmacotherapy, as well as encouraging the treatment of all possible coexisting COPCs, may improve clinical outcomes in these patients, overcoming the need to prescribe second-line therapies, or to resort to surgery [9]. Accordingly, the identification and quantification of the central component of pain may identify patients who will fail to respond to surgery. This may entail both clinical and legal consequences [10].

For these reasons, the literature regarding the use of tools for the detection CS in patients with endometriosis is increasing. However, there is no gold standard measure for CS and available tools include both self-reported questionnaires and objective measures such as QST, neuroimaging techniques, and somatosensory-evoked potentials. While the latter are considered complex, expensive, and lengthy, and as such are more frequently used in research settings, the former, and especially the CSI, may represent a valid aid in clinical practice [15].

The fact that the CSI was the most frequently used questionnaire among the studies included in our review is probably due to it having been specifically validated in patients with endometriosis [6]. Also, its good psychometric proprieties have been proven in a greater number of studies, compared to the FSQ. Moreover, the CSI has been found to respond to treatment, both in chronic pain populations and in patients with endometriosis [6, 33, 44], while responsiveness of the FSQ is yet to be proven. The CSI is also provided with a specific cut-off value, and various attempts have been made to establish clinically relevant CSI severity levels [33, 63].

The FSQ was initially intended to be used in patients with fibromyalgia and as such does not investigate the presence of other COPCs as extensively as the CSI [34]. The increasing recognition of a central component to fibromyalgia-related pain has led to its application in other centrally derived chronic pain conditions; however, at the present time, its use is still scant.

One of the main characteristics of the FSQ is that it does not have a cut-off value for CS. This may appear a downside of the questionnaire; however, it reflects the hypothesis that features of fibromyalgia—and consequently of CS—extend to individuals who do not satisfy the criteria for fibromyalgia, or for any other COPC [34]. In fact, according to Hauser and co-workers, fibromyalgia is a clinical entity at the end of a continuum of biopsychosocial distress, which may be defined as “fibromyalgianess” [50]. Similarly, Mayer and colleagues found that the symptoms investigated in the CSI occur “sometimes” in most individuals [31], while Neblett and co-workers stated that “subclinical” CS is present in many healthy controls [33], reinforcing the concept that CS is detectable in all individuals, although with variable grades of severity. According to Neblett and colleagues, patients with “subclinical” CS should be monitored over time, as they may be more prone to developing a COPC in the future, especially if they have a history of abuse and/or psychiatric disorders [33].

In conclusion, despite an increasing use of these self-reported questionnaires for the measurement of CS, the debate regarding their construct validity is still open. In fact, although theoretically a gold standard diagnostic tool is essential to establish the construct validity of questionnaires designed to screen for a given feature, such gold standard tool for the diagnosis of CS is not yet available [43]. The authors argue that questionnaires measuring CS quantify the entity of COPC-related symptoms, although the hypothesis that COPCs are centrally driven is still only theoretical. In their recent meta-analysis, Adams and co-workers found that the correlation between the CSI and various quantitative sensory tests for CS was weak, negligible, or even absent (*r* = − 0.2 to 0.1), while that between the CSI and psychological questionnaires for anxiety, depression, catastrophizing, stress, sleep, and kinesiophobia was moderate to strong (*r* = 0.4–0.6) [64]. Thus, in the few past years in which it has started to gain attention, the term “central sensitization” seems to have undergone a construct drift from its preclinical meaning of enhanced responsivity of central nociceptive neurons to a broader connotation including psychological status [64] and the CSI seems to identify individuals with a psychological vulnerability and a hypervigilant state that is associated with pain, rather than CS itself.

This caveat is not resolvable until the debate regarding the definition of CS is set aside by the establishment of a set of defining criteria for this phenomenon. Arguably, CS-related symptoms may be causative, a consequence, or even a coincidence in patients with chronic pain [30]. However, whatever the relation between CS and chronic pain, tools for the detection of CS are revealing the existence of a part of the population which warrants further attention, both from a research and from a clinical point of view. In our opinion, this is a sufficient reason to encourage their use in patients with endometriosis.

## Conclusions

The CSI is the most frequently used questionnaire for the detection of CS in patients with endometriosis. Probably this is due to the fact that it has been specifically validated in this population and that it has been found to have good psychometric proprieties. Although it was originally intended to be used in patients with fibromyalgia and its psychometric proprieties have been studied less extensively, the FSQ has also used to screen for CS in individuals with endometriosis. Further research is needed to better comprehend construct validity of both questionnaires, as a gold standard diagnostic tool for CS is currently not available. However, their use should be encouraged both in research and in clinical settings as they are able to identify chronic pain patients who may benefit from a broader treatment strategy, which includes but is not limited to conventional therapies.

### Supplementary Information

Below is the link to the electronic supplementary material.

ESM 1 (32.3 KB docx)

## Data Availability

On demand.
